# Credibility judgments in web page design – a brief review

**Published:** 2016

**Authors:** O Selejan, DF Muresanu, L Popa, I Muresanu-Oloeriu, D Iudean, A Buzoianu, S Suciu

**Affiliations:** * “RoNeuro” Institute for Neurological Research and Diagnostic, Cluj-Napoca, Romania; ** Department of Neurosciences, “Iuliu Hatieganu” University of Medicine and Pharmacy, Cluj-Napoca, Romania; *** Department of Clinical Pharmacology and Toxicology, “Iuliu Hatieganu” University of Medicine and Pharmacy,Cluj-Napoca, Romania; **** Faculty of Electrical Engineering, Technical University of Cluj-Napoca, Cluj-Napoca, Romania; ***** Department of Functional Biosciences, “Iuliu Hatieganu” University of Medicine and Pharmacy, Cluj-Napoca, Romania

**Keywords:** user credibility, web page aesthetics, visual hierarchy, web page perception

## Abstract

Today, more than ever, knowledge that interfaces appearance analysis is a crucial point in human-computer interaction field has been accepted. As nowadays virtually anyone can publish information on the web, the credibility role has grown increasingly important in relation to the web-based content. Areas like trust, credibility, and behavior, doubled by overall impression and user expectation are today in the spotlight of research compared to the last period, when other pragmatic areas such as usability and utility were considered. Credibility has been discussed as a theoretical construct in the field of communication in the past decades and revealed that people tend to evaluate the credibility of communication primarily by the communicator’s expertise. Other factors involved in the content communication process are trustworthiness and dynamism as well as various other criteria but to a lower extent. In this brief review, factors like web page aesthetics, browsing experiences and user experience are considered.

## Aesthetics and credibility in web page design

Aesthetics has largely been assessed by means of a single bipolar item (e.g. ugly–beautiful), which reflects a gut feeling at best but not a profound aesthetic judgment. However, this simple and intuitive appraisal can be very useful and has its justification in a quick assessment of first impressions, as it was demonstrated by Lindgaard, Fernandes, Dudek, and Brown [**1**]. The authors were able to show that the first impression of a website is formed within 50ms and is highly stable. Moreover, it can be seen as the most prototypical aesthetic judgment. This 50ms window is certainly not enough time for cognitive processes to occur in an analytical or reflective manner but it showed that ‘‘visual appeal’’ was the prime determiner of a positive reaction to a website. This very short time span is aligned with a result of another study, in which authors were arguing that 80% of the people browsing the web spend just a few seconds on a site before moving along [**2**]. 

Even if there are many best practice guidelines for aesthetic design, the body of knowledge is still looking for more solid data about empirically validated user interface (UI) design factors. It is of utmost importance to categorize the triggers of the users’ aesthetic responses. According to Michailidou et al. [**3**], the ‘‘less is more” notion, showing that less complex websites are preferred over more complex ones, was found valid. Robins and Holmes [**4**] argued, in the same vein, that when a person is opening a website, the first impression is probably made in a few seconds. Based on this first impression, the user will either continue the browsing or move on to the next web page, a decision influenced by many factors. 

Page aesthetics and user’s judgment about the site’s credibility is among the factors that may influence the user to continue its browsing on a web page or go away. A study performed by Rieh & Danielson [**5**] showed that when an identical content is delivered to users using different levels of aesthetic treatment, the page with a higher aesthetic treatment was judged as having higher credibility. The authors coined that terms such as the “amelioration effect” of visual design and aesthetics on content credibility – an aesthetic treatment, increased the rating for the same content in 19 out of 21 cases (90%). In the first few seconds in which a user views a web page, this effect is already settling in. As depicted before, in a case of content similarity, a higher aesthetic treatment will increase perceived credibility. An important aspect to be mentioned here is that credibility can take different forms in the eyes of the users. Some of them will perceive content quality on a website as a source of credibility while others can perceive authority as a sign of credibility in the online environment [**6**]. A web page logo is also seen as an authority sign. The term “credibility” is used here to describe the extent to which users trust the informational content on a certain website.

Fogg et al. [**7**] conducted extensive studies on the phenomenon of web credibility that revealed surprising results on the extent to which the dynamism of a website mattered to users. The largest category, “design and look”, was indicated by 46.1% of the respondents. The second preferred category was “information design” of a site and was indicated by 28.5% of the respondents as a marker that contributed to their credibility judgments. To summarize, nearly 75% of the respondents reported making credibility judgments by content presentation rather than other factors (content’s/ creator’s authority, trustworthiness, reputation, etc.).

## The layers of credibility judgments

Norman [**8**] suggested that credibility judgments might occur at different levels of perception and criteria, classified as visceral and cognitive. He divided reactions to design in three experience levels: visceral, behavioral, and reflective. Visceral experience in design is an immediate, powerful reaction to design while the behavioral level represents the experience during the use of design. Whereas the visceral design tries to capture the user’s attention immediately, the behavioral design aims to keep the user focus on the page through the ease of use and learning. However, it may represent the fact that users will transcend the behavioral level and use objects that do not perform well because of some emotional attachment to the object. This represents the reflective level. The design in this area is highly analytic and cognitive and an attempt to create a better design by incorporating the experience of users and their knowledge of goals and objectives of the product or service is made [**9**]. 

The ‘‘visceral’’ criterion represents an area in which a reduced number of studies have been conducted. Viscerally-based credibility judgments emerge without conscious analytical cognitive processes. This reaction is primarily based on highly subjective reactions to stimuli presented when a user starts browsing a website. In this train of thoughts, a person’s credibility judgment may be influenced by a combination of different factors (e.g. colors, layout, fonts, bulleted lists, tabular data, etc.). The users will find the task of explaining these judgments challenging. They usually relate to such factors as dynamism, trustworthiness (if based on intangible factors such as first impressions), and sociability. 

Anyhow, viscerally influenced criteria are primarily visual and not cognitive, so the impact of the visual experience is an action facilitated at the level of the nervous system and not at the level of brain thought processes. Gladwell [**10**] and colleagues summarized the research on rapid cognition, while Wathan and Burkell [**11**] presented a similar notion in their model of the credibility judgment process. These studies tried to explain how people can make quick judgments that are often correct. The authors identified cognitive processes like ‘‘surface credibility’’ (visceral) and ‘‘message credibility’’ (cognitive). The latter requires a further analysis to evaluate more objective criteria (e.g. expertise, accuracy), while the former addresses appearance issues that were quickly processed.

If at the visceral level, the design of a website suggests that the information is not credible, the viewer might decide to leave the page after a very short period of time, thus not allowing the content credibility to be perceived and judged at the cognitive level.

Tractinsky et al. [**12**] designed two experiments to replicate and continue Lindgaard’s work. By using explicit (subjective evaluations) and implicit (response latency) measures in both experiments, they have demonstrated that immediate aesthetic impression of web pages are remarkably consistent. In the first experiment, the participants evaluated and ranked the attractiveness of 50 web pages in two phases after two exposures: 500ms and 10 seconds. The ratings of web pages after the 500ms were strongly correlated with the average attractiveness ratings after a 10 seconds exposure. The findings also suggested considerable individual differences in evaluations and the consistency of those evaluations.

In the second experiment, the same 500ms exposure was preferred for 24 of the 50 web pages from the first experiment. The same marker was evaluated as in the first study: attractiveness. Subsequently, users evaluated the design of the web pages on the dimensions of classical and expressive aesthetics. The results showed a high correlation between the attractiveness ratings on both experiments. Also, it seemed that low marks in attractiveness were mainly associated by subjects with very low ratings of expressive aesthetics. Overall, the main conclusion is that aesthetic impressions of web pages are quickly made and these results provide direct evidence in support of this premise. Indirectly, these results also suggested that visual aesthetics play an important role in the users’ evaluations of the IT artifact and their attitudes toward the interactive systems.

## The model of visual hierarchy 

The mind-eye hypothesis implies that people are usually thinking about what they are looking at [**13**]. They do not always totally understand or engage with it, but if they are looking, they are usually paying attention, especially when concentrating on a particular task [**14**]. 

The mind-eye hypothesis also implies that the way people look at any given artifact (e.g. web page) is determined by what they are trying to do with it. In other words, the task the user has chosen or been asked to do determines their looks [**13**].

As presented by Faraday [**15**], the viewing pattern is guided by two distinct cognitive processes: searching (a process that can be determined by vectors such as text style, color, size, location and visual information of components) and scanning (driven by attributes such as proximity and order of components). Searching refers to a viewer’s attempt to find a point of entry into the page while scanning refers to the viewer’s behavior after finding such an entry point. In this second phase, the viewer extracts information that is located at the entry point. As larger items draw more attention than smaller items, larger objects on a page will be viewed prior to the smaller ones [**16**]. People also exhibit a top down viewing preference. Therefore, items located at the top of a page will have priority in the visual hierarchy over other items. The scanning phase of viewing can also be influenced by items nearby, which are perceived as related to each other. Placing related information around an entry point on a web page can facilitate a more effective scan phase [**17**].

Interesting findings on reading preferences of long documents were published by Buscher et al. [**18**]. This exploratory study analyzed reading regions on a monitor. The authors have proved that the users’ visual attention was not evenly distributed on the screen and that users have individual preferred reading regions when working with long documents. Vertically, the visual attention can be approximated by a normal distribution specified by two parameters: the preferred vertical reading location and the amount of vertical spreading. 

A survey published by Nielsen [**19**] indicated a return on the investment as high as 83% in websites in which users defined their browsing experiences as positive. More than that, if the page is visually pleasing, users are more inclined to trust it [**20**]. In the same vein, the visual appeal of a page is positively correlated with a perception of usability [**21**]. 

Djamasbi et al. [**22**] conducted two studies, trying to confirm the prior mentioned hypotheses, in which they compared the users’ opinion on two web pages. As a methodology, they used two prototypes of the same page to examine if including images of people had an influence on perceptions of visual appeal and whether a user’s trust assessment was correlated with the visual appeal rating. The results showed that the page with images of people was rated significantly more visually appealing than the page that included images of logos. These results are consistent with the social presence theory and suggest that the inclusion of images can positively affect the appeal of a homepage. Moreover, the investigator found that the participants’ visual appeal ratings were found to be a significant predictor of their credibility rating and that the people completed tasks significantly faster by using the page with images of people while maintaining the accuracy. These results support the literature suggesting that the beauty of a page may affect people’s trust in it [**23**]. Another study conducted by Cry et al. [**24**] reinforced these findings: pages that include human faces are perceived to have a greater degree of social presence. 

Nevertheless, Djamasbi’s findings are not in line with the results published by Lewenstein et al. [**25**]. In a study in which users were examined based on the way they read online news articles, the authors measured their first three gazes on a page. The results indicated that the users’ attention was drawn to text over graphics and photos, and ran against findings from traditional print media that suggested that users are attracted by photo elements first.

## Gender differences and age in web pages perception

Starting from the evidence presented by Moss et al. [**26**], arguing that men and women exhibit different preferences in layout and presentation stimuli, Djamasbi and his colleagues examined possible gender differences in web preferences by using eye tracking [**16**]. Literature provides ample evidence that men and women exhibit differences in what they perceive as attractive and when designing websites. Also, men and women tend to show different preferences in how they create their web pages regarding several factors [**26**]. The same study revealed that men prefer to use darker colors (e.g. black, blue) compared to women, who prefer lighter colors. Also, women are more prone to include images in their web design [**26**]. In particular, women are more prone to include images of people in their websites compared to men.

In a study performed on 30 subjects, Pan et al. [**27**] investigated the determinants of web page viewing behavior by using eye-tracking. They have concluded that the gender of subjects drives the web page viewing behavior, the order of web pages viewed and the interaction between site types and the order of the pages viewed. Some important results of this study revealed that males exhibited significantly longer mean fixation durations than females. Gender differences in perceptual processing have also been reported by Jones et al. [**28**]. The gender differences reported in the current study provided further support for the notion that different design guidelines might be beneficial to websites who cater specifically to one gender or the other [**27**].

Nevertheless, the most interesting finding is the complex interaction effect of page order and site type, on the three measurements of ocular behavior, meaning that the viewers’ eye movement behavior changes over time even on a single website, and the type of websites influenced the change in direction and magnitude. This confirms the previous work of other researchers, supporting the hypothesis that the individual characteristics of the viewer, as well as the stimuli, contribute to the viewers’ eye movement behavior [**29**]. 

In order to close the circle of age and gender, Djamasbi et al. [**16**] performed a study in which Generation Y’s [**18**-**30**] web preferences were investigated. This population segment spends 200 billion dollars per year and represents a significant market share per se. Regarding business and practical implications, this study has proved to deliver important conclusions, and that is because Generation Y has very solid internet skills that are averse to irrelevant marketing [**30**]. In complementary studies related, authors found out that Generation Y people like cool graphics, have short attention span, and do not like to read long boring texts. It is more likely that this generation particularly enjoys the presence of images on web pages [**30**]. 

The results of this study were in line with the prior research that showed people under forty like pages that provide a search feature, include pictures of celebrities, have little text, and contain a large main image.

## Conclusions

The credibility study is highly multidisciplinary and it involves some different concepts and approaches, spanning from information evaluation, content quality, page aesthetics, and gender preferences, etc. In this brief review, our work has focused on the cognitive process involved in the credibility evaluation of a web page, the content impact on this perception as well as on the divided preferences between genders. 

As depicted by Popa et al. [**30**], there are several tools for the exploration of cerebral processes, and we can mention the following: eye tracking, functional magnetic resonance imaging, brain–computer interface, human–computer interaction, e-learning, and assistive technology. Hopefully, shortly, the study of credibility will benefit from this complex array of options hence revealing new insights in the particular field.
